# Research on dye sensitized solar cells: recent advancement toward the various constituents of dye sensitized solar cells for efficiency enhancement and future prospects

**DOI:** 10.1039/d3ra00903c

**Published:** 2023-06-28

**Authors:** Sultana Rahman, Abdul Haleem, Muhammad Siddiq, Muhammad Khalid Hussain, Samina Qamar, Safia Hameed, Muhammad Waris

**Affiliations:** a Department of Chemistry Quaid-i-Azam University 45320 Islamabad Pakistan haleem0300@gmail.com m_sidiq12@yahoo.com; b Department of Physics, Faculty of Science, University of Gujrat HH Campus Gujrat 50700 Pakistan; c Department of Physics, Faculty of Science, University of Gujrat, Sub-Campus Mandi Bahauddin 50400 Pakistan; d Department of Information Engineering University of Brescia Italy; e National Centre of Excellence in Analytical Chemistry, University of Sindh Jamshoro 76080 Pakistan

## Abstract

It is universally accepted that the financial advancement of a state is essentially dependent upon the energy sector as it is essential in the growth, development, and improvement of the farming, mechanical, and defense sectors. A dependable source of energy is expected to enhance society's expectation of everyday comforts. Modern industrial advancement, which is indispensable for any nation, relies upon electricity. The principal explanation behind the energy emergency is rapidly increasing the use of hydrocarbon resources. Thus, the use of renewable resources is essential to overcome this dilemma. The consumption of hydrocarbon fuels and their discharge has destructive consequences on our surroundings. Third-generation photovoltaic (solar) cells are latest encouraging option in solar cells. Currently, dye-sensitized solar cells (DSSC) utilize organic (natural and synthetic) dye and inorganic (ruthenium) as a sensitizer. The nature of this dye combined with different variables has brought about a change in its use. Natural dyes are a feasible alternative in comparison to expensive and rare ruthenium dye owing to their low cast, easy utility, abundant supply of resources, and no environmental threat. In this review, the dyes generally utilized in DSSC are discussed. The DSSC criteria and components are explained, and the progress in inorganic and natural dyes is monitored. Scientists involved in this emerging technology will benefit from this examination.

## Abstract 2

It is universally accepted that the financial advancement of a state is essentially dependent upon the energy sector as it is essential in the growth, development, and improvement of the farming, mechanical, and defense sectors.

## Graphical abstract

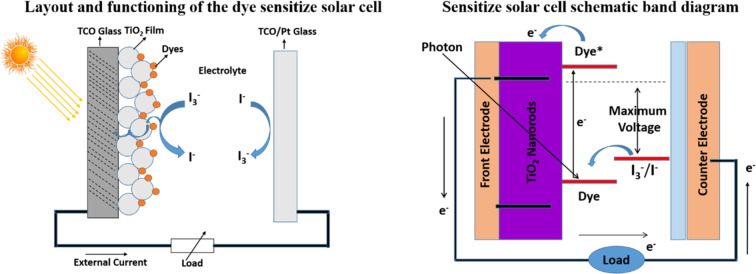

## Introduction

**Fig. 1:**
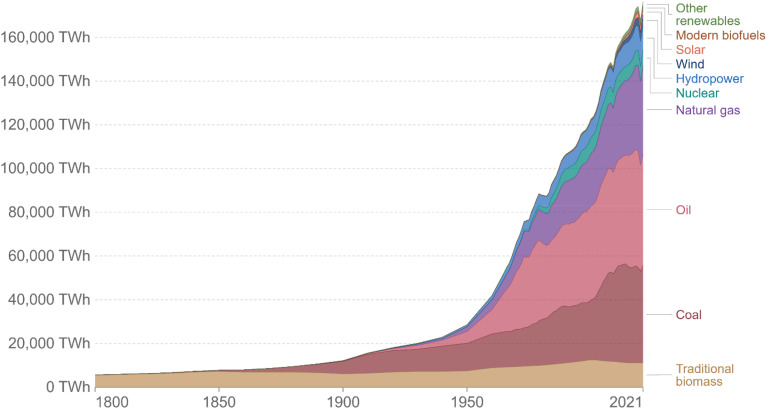
Source: our world in the data based on Vaclav Smil (2017) and BP statistical review of the world energy.

In recent times, energy generation has become an essential scientific and technological need. The field of renewable energy is one of the many pursued areas of investigation and, expectedly, it shall continue to be so for several decades in the future. Easy access to water and energy sources is neither extensive nor has it been effectively accessible for more than 50 years to individuals in some nations and metropolitan regions around the world. The term “renewable energy” refers to energy obtained from a wide range of resources that all rely on self-renewing energy sources, such as sunshine, wind, flowing water, Earth's internal heat, biomass-containing energy crops, agricultural and industrial waste, and municipal trash. These sources can be used to generate electricity for all sectors of the economy, fuel transportation, and heating for buildings and industrial processes. Power energy and water accessibility tend to be entwined; if one faces an emergency, the sufficiency and safety associated with the other is likewise profoundly influenced. Moreover, currently, both are in high demand. Therefore, it is urgent to deal with the worldwide increase in energy demands, and, in the meantime, diminish greenhouse gas emissions to limit global warming. Since the Industrial Revolution, the energy sector has undergone significant changes. The interactive chart given in Fig. 1 illustrates how the world's energy supply is changing, displaying a graph of world energy use starting from the 1800s onwards based on historical primary energy consumption projections from Vaclav Smil and current data from BP's Statistical Review of World Energy.^1^

Under sensible presumptions of populace development and power generation, the projection for the power demand worldwide in 2050 is 28 terawatt (TW).^2,3^ Solar power has the greatest potential to fulfil the requirement of renewable power sources in the near future globally. A helpful Earth-bound global worldwide solar power prospective estimate is assessed at about 600 TW through the 1.7 × 10^5^ TW solar energy that strikes the surface of our planet. Therefore, making use of 10% productive ranches that are sunlight-based, about 60 TW of energy could possibly be provided. On the other hand, solar power cell creation has developed at about 30% per year in the last 15 years.

**Fig. 2:**
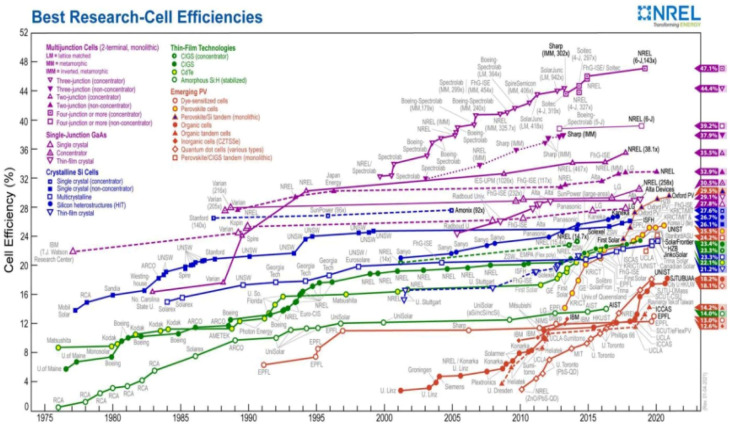
Year-by-year status of the efficiencies of different PV technologies. Reproduced with permission from National Renewable Energy Laboratory, Golden, CO.

A concise summary of global research on various DSSC components prior to going into the research and development efforts made for the various DSSC components has been given below, dealing with some salient works on the international status of DSSC research advancement. Fig. 2 illustrates the photoconversion efficiency of several types of solar cells, including DSSC, based on NREL (USA) year-by-year analysis from 1980 to the present. It is noteworthy to highlight that over the past 20 years, no material has been found to increase the efficiency of DSSCs, though PSCs have become promising photovoltaic technologies in a relatively short time of just one decade. We will discuss about a few significant research and innovation initiatives that have been carried out globally on the various DSSC components during the past ten to fifteen years. According to the analysis, most research efforts have been directed at creating DSSCs that are affordable, ecofriendly, stable, and effective. The most researched photoanode materials for DSSCs are still TiO_2_ and ZnO. A few TiO_2_-modified photoanodes, such as SiO_2_/Ag/TiO_2_,^4^ g-C_3_N_4_ and ZnO/TiO_2_,^5^ multilayer TiO_2_,^6^ Ag/TiO_2_,^7^ Nb/TiO_2_,^8^ CuO/TiO_2_,^9^ and carbon black-TiO_2_,^10^ have demonstrated promising photovoltaic capabilities. Mn/ZnO,^11^ Cu/ZnO,^12^ ZnO/TiO_2_ nanocomposite,^13^ ZnAl-MMO/graphene,^14^ ZnO/MWCNT,^15^ Al/ZnO,^16^*etc.*, are also promising ZnO-based photoanodes, demonstrating promising electron transport in the photoanode and increased cell efficiency. On exposure to light, dye molecules generate photoexcited electrons, and are thus regarded as the most crucial part of DSSCs. Organometallic dyes based on ruthenium are the most effective dyes for DSSCs (N3, N719, N749, *etc.*).

The traditional solar cell of today, the first-generation solar cell, depends on silicon.

The considered installation limit in 2007 had been 7.8 giga watt (GW).^17,18^ In 2008, the photovoltaic, a global organization, expanded by 6.0 GW. However, the photovoltaic (PV) industry is to an extent that is surely great on legislative endowments.^19,20^ PV systems already provide 1.7% of the gross electricity production given in Organization for Economic Co-operation and Development.^21^ Currently, silicon-based frameworks comprise about 90% of this PV case. According to International Energy Agency, photovoltaics (PV) is the energy technology with the fastest growth and should pass the 300 GW global installation mark in 2017.^21^ This rapid evolution illustrates the complementarity of fundamental research, dedicated to reaching highest performances and industrial developments, thus turning laboratory results into commercial systems. The creation cost is about $3 perW p; however, it is remarkably subject to the cost of silicon material. China is currently the main worldwide producer of crystalline silicon (c-Si)-based PV cells and segments by way of a limitation of over 2.3 GW per year. The International Energy Agency (IEA) has evaluated the power recompense period of c-Si PV segments, combined like a lattice-connected roof establishment that is top as somewhere in the product range of 1.5 and 2.5 years. The second-generation solar cells, for instance, amorphous silicon, CIGS, and Cd Te, depend on thin film advances. The upsides of thin layer solar cells integrate the ease of fabricate, allowing a decrease in the generation price to about $1 per W p (watt peak), an appealing scope, and conceivable results of using adaptable substrates. The essential settled thin-film innovation is amorphous silicon, *i.e.*, (a-Si).^22,23^ The effectiveness is smaller than c-Si; however, it offers various situations that are favorable as well as a reduced temperature coefficient for energy loss. The cost is just marginally less than that compared to c-Si for the component that is most important in expensive assembling apparatus. Through these decisions, solar power addresses an amazing appealing option to fulfill our energy requirements later, considering the method in which the power conveyed from the sunshine is all-natural and abundant. In the past, DSSCs have risen as a viable alternative to traditional solar cells due to their simplicity and ease of production. Silicon-based solar cells cover over 80% of the world installed capacity today^24^ and currently represent 90% of the market shares.^25^

## Solar energy

**Fig. 3:**
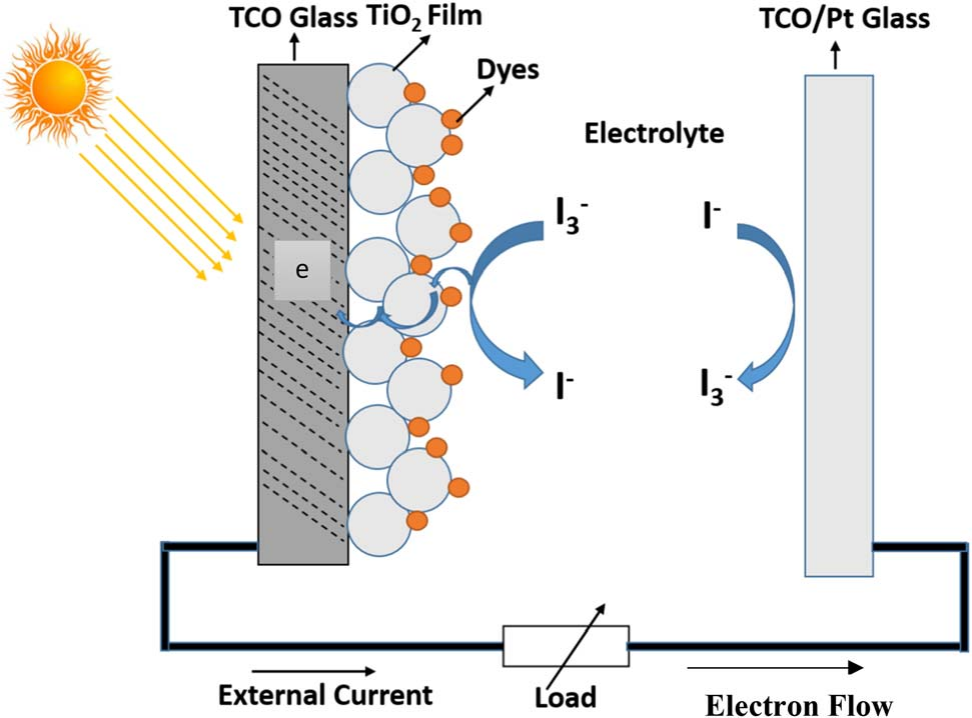
Layout and functioning of the DSSC diagram.

Solar energy is the quickest-rising renewable energy technology having the potential to meet a large portion of future global energy demand.^26^ DSSC are devices or instruments for the transformation of visible light into electricity and have pulled in much consideration as easy photovoltaic cells and turn into a quickly growing technology with efficient application. The current DSSC construction involves a set of different layers of components, as shown in Fig. 3, which includes the glass substrate, transparent conducting layer, TiO_2_ nanoparticles, dyes, electrolyte (I^−^/I^3−^), and counter electrode. In DSSC, the dye used as a sensitizer has an important task in retaining sun light as well as changing sun-powered energy into electric energy. DSSCs or photoelectrochemical cells have been oppressed of an expansive wide range of research examinations since 1991. DSSCs isolate the optical absorption and charge partition procedure by partnering a sensitizer with a large band gap semiconductor of nanocrystalline shape. As indicated by Y. Chiba *et al.*, the total conversion efficiency was recorded as 8.12%, 10.10%, 10.40%, and 9.90% and reported at Energy Research Centre of the Netherlands, Ecole Poly Technique Federal de Lausanne, and Sharp Corporation and Arakawa, individually for DSSCs with aperture area of 1–5 cm^2^. Obviously, the utilization of high film titanium dioxide cathodes prompted the most elevated productivity till date, remaining at 11.10%.^27^ Ruthenium-based complexes sensitizers have been broadly utilized because they have better efficiency and high strength, and these focal points are possibly balanced by their surprising expense, their entangled manufactured courses, and the inclination to experience degradation in the presence of water. Likewise, noble metals are considered as assets that are constrained in amount, and their production is exorbitant. Endeavors are consistently being embraced to enhance the execution of DSSCs and thus the intensity of this innovation on the world market. DSSCs can be viewed as an innovation between the second and third era devices. This can possibly turn into a third era innovation using the nanoscale properties of the devices. In the present stage, the innovation suggests the accompanying moving focuses.

• Especially fascinating much lower investing price contrasted and ordinary PV advancements.

• Type of opportunities, for example, clarity and multicolor alternatives (building incorporation, customer items, and so on.)

• Versatility.

• Easily portable/lightweight.

• Feedstock availability to accomplish TW scale.

• Fast energy recompense time (<1 year).

• Improved execution under genuine outdoors situations (more advanced than contenders at diffuse light and more temperatures).

• Bifacial cells catch light from all edges.

• Outclass competitors for exclusive application.

## Material challenges for the twenty-first century in DSSCs

We addressed the state-of-the-art in photovoltaics as well as the remarkable development over the past few decades. There are still a lot of obstacles to overcome before photovoltaic energy can account for a sizable portion of the world's energy production, despite having roughly 300 gigawatts of installed peak power and possibly reaching 2% of it very soon.

(i) Efficiencies are still much below what thermodynamics permits. Theoretically, photovoltaic systems can convert more energy than 33% of solar energy into electrical energy for single connections and potentially up to 90% if the right materials can be found. Materials for tandems and innovative conversion technologies such as intermediate band or hot-carrier solar cells are particularly important. Efficiencies are particularly sensitive to chemical and structural flaws, even at low concentrations, and highly reliant on the caliber of the materials used.

(ii) The availability of materials and their tendency for low-cost processing. If highly scalable and affordable procedures (such as printing technologies) could be employed to create high-quality materials, extremely low cost would be possible. Over the past ten years, the supply of materials has also become a factor as questions about the long-term viability of the technology are raised by the expansion of manufacturing.

(iii) Material ageing and durability at the solar cell and module levels are also a problem because they have an impact on the technology's dependability and, ultimately, cost. The intrinsic stability of the active materials was frequently found to be a problem that needed to be solved first and contributed to the failure of various technologies in the past, such as Cu_2_S/CdS.

(iv) As the production numbers (the TW scale) increase and supply chain issues, particularly environmental ones, come to the forefront, life cycle limits (toxicity, recyclability, including structure materials) may become more frequent. These challenges are being addressed in an increasing number of studies.

(v) Integration with the global energy system (system, storage) and the built environment (storage, aspect), which are currently hot topics in the penetration of energy production (2% of electricity), which is close to the point where power management is critical, is taken into consideration. Again, more applications are available due to increased prices and improved performance.

Most of the challenges mentioned above will probably demand completely new approaches. While current performance levels (including reliability and efficiency) and competitiveness have been enough to reach a detectable penetration in energy production, new performance levels will be needed to make a major contribution to this mix. Performance has an effect on costs in addition to favorably influencing sustainability. Environmental footprint and life cycle issues will become more significant when measured in terawatts. For these reasons, we have decided to concentrate on new strategies in this topic that could assist in resolving these problems. We shall first think about possible approaches to the efficiency frontier. We will then talk about novel materials, particularly molecular, colloidal, or hybrid materials, for greater integration and solar competitiveness. Finally, we will provide some examples of how novel material used in the device and their characterization are essential for the emergence of the aforementioned developing technologies.

## Structure and principle of DSSCs

**Fig. 4:**
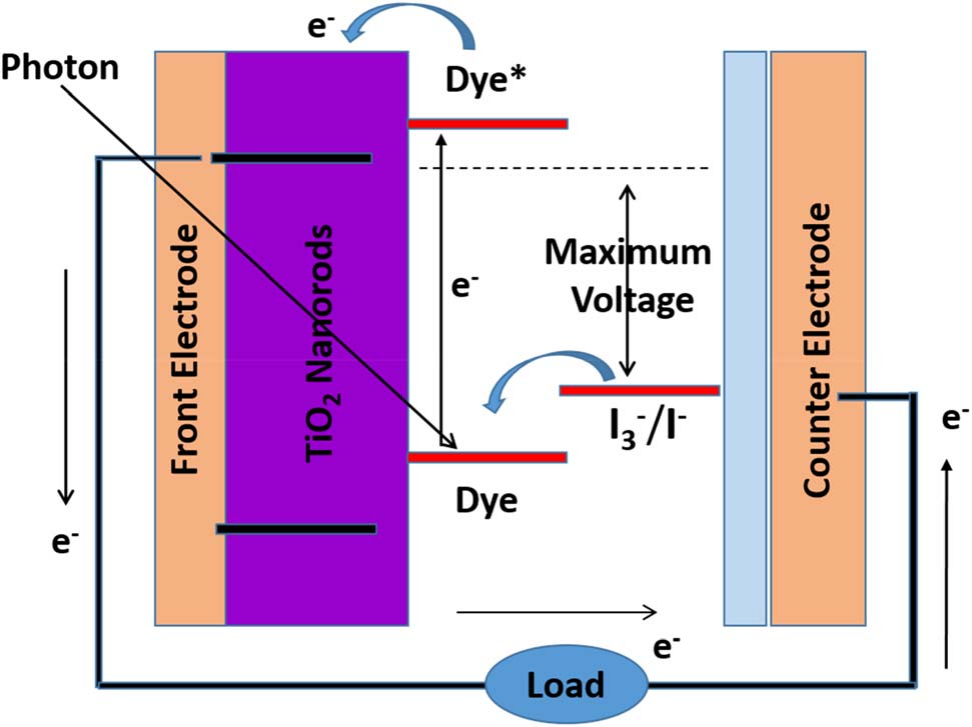
Sensitize solar cell schematic band diagram.

A schematic depiction of DSSCs is shown in Fig. 4, where the framework is made from four fundamental parts.

(I) A photoanode fabricated from a mesoporous oxide layer (commonly, titanium dioxide) kept on a transparent conductive glass substrate.

(II) The dye monolayer covalently attached to the surface of the titanium dioxide layer for light harvesting and produces electrons that are photon energized.

(III) In an organic solvent, the electrolyte having the I^−^/I^3−^ as the redox couple together with the electron at the counter electrode and the recovery of the effecting sensitizer.

(IV) Conductive glass substrate fully covered by platinum, which made the counter terminal.

## Operation of DSSCs

**Fig. 5:**
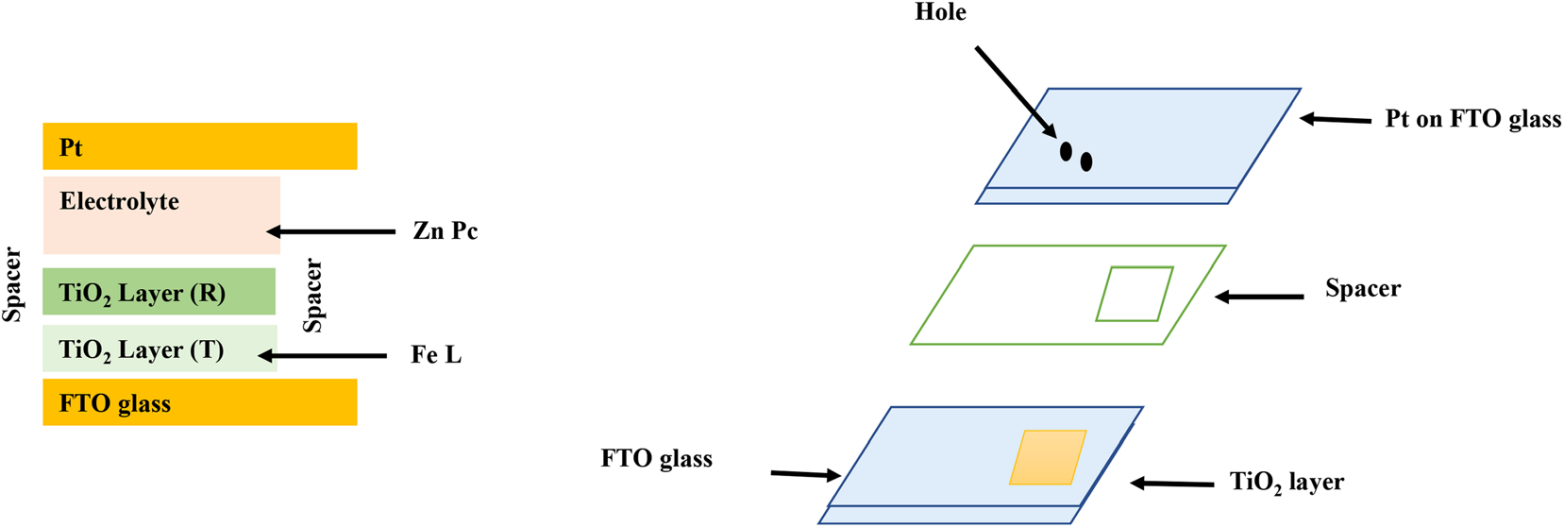
(Left) Structure of a DSSC (Right) photograph of the DSSC package.

The architecture of DSSCs consists of two main parts, one of which is platinum-doped or graphite-plated working electrode, and the other of which is a thin crystalline semiconductor with a large band gap (such as titanium dioxide) sensitized with a complex dye (Fig. 5). An electrolyte solution arrangement covers the distance between two electrodes. The fundamentals of DSSCs include certain essential procedures such as the assimilation of light, departure, and accumulation of charge. The dye will retain photons and proceed toward being photoactivated when exposed to sunshine. The ingested sensitizer particles shall infuse electrons into the titanium dioxide working terminal and, in this way, be oxidized. Partition of charge attain over the interface of semiconductor, where in titanium dioxide an electron is located, and a gap is placed in the oxidized dye particle. The electrons will then pass through the titanium dioxide permeable system and eventually reach the working electrode's back contact, where the accumulation and extraction of charge will occur. The removed charge can perform electrical work in the outer circuit in this manner and finally come back to the counter cathode, where a decrease in the redox mediator happens. The oxidized dye will be integrated into the circuit by the liquid redox electrolyte.

To accomplish the light harvesting capacity by the dye sensitizer in the DSSC, there are certain qualities that exhibit the helpfulness of the compound to be utilized or not. In addition, the dye sensitizer can affect enormous quantities of the exchange of the main electron takes place at the interface of titanium dioxide/dye/interface of the electrolyte, which, moreover, chooses the productivity of the created device. The structure of the dye can affect and drive electron infusion forward into the titanium dioxide conduction band. Then again, the structure of the sensitizer helps hinder electron assign procedures. In earlier DSSC investigations, the conventional dye N3 and N719 as salt of N3 and the dark dye N 749, which is among the most important components of dye-sensitized photovoltaic cells (DSSCs), must meet a few basic requirements.

(1) The photosensitizer's absorption band must encompass the full visible spectrum, as well as a portion of the adjacent infrared spectrum (NIR).

(2) Anchoring units (–COOH, –H_2_PO_3_, –SO_3_H, *etc.*) should be included in the photosensitizer to keep the dye firmly attached to the surface of the semiconductor.

(3) The photosensitizer's energized phase dimension must be greater in energy than the n-type semiconductor's conduction band edge (n-type DSSCs) for a productive electron transfer activity between the energized dye and the semiconductor's conduction band (CB).^28^ In p-type DSCs, the photosensitizer's HOMO dimension should be at a higher positive potential than the p-type dimension of the semiconductor valence band (VB).

(4) For sensitizer recovery, the photosensitizer's oxidized form ought to be greater than the redox electrolyte capability.

(5) Adverse dye accumulation on the surface of the semiconductor should be avoided by improving the dye's subatomic structure or by expanding the co-adsorbent that inhibits agglomeration. The sensitizer can be regulated in either situation (H- and J-totals), resulting in improved performance as compared to a monomer dye layer.

(6) The photosensitizer must be photostable, and it must also be electrochemically and thermally secure. A wide range of photosensitizers, including metal-free, natural dyes,^29^ porphyrins, and phthalo cyanines, have been constructed and connected to DSSCs in recent decades in response to these requirements.

(7) Research is currently being done to enhance dye designs that can not only provide high power conversion efficiency but also have a greater capacity to scale above the limit of 19 GW per year, the limit set by the accessibility of ruthenium.^30^ The development of effective organic sensitizers is currently taking place. To create novel organic dyes that are more stable and effective, new structural engineering efforts are being made with donor–pi–acceptor, or (D–π–A) 2, dyes. Thiophene units were used as the pi-bridge, branching (D–π–A) comprising stiff alkyl-functionalized carbazole core as the donor part, and cyanoacrylate moiety as the acceptor and anchoring portion. The highest power conversion efficiency reached 5.01%, 0.70 V, and photocurrent of 10.52 mA cm^−2^.

**Fig. 6:**
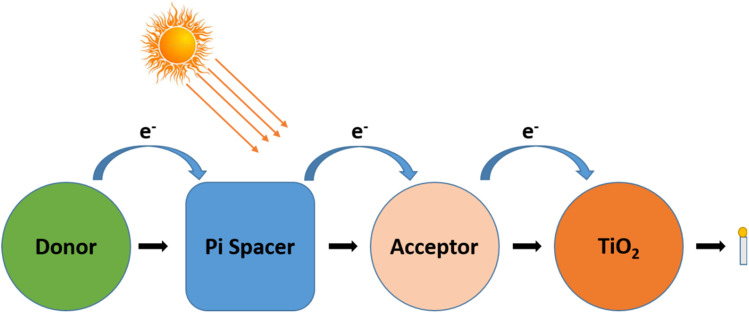
D–π–A (donor–pi–acceptor) dye structure and its functioning in DSSCs.

Fig. 6 depicts a D–π–A (donor–pi–acceptor) sensitizer structure. The process underlying the impact of conjugation in the sensitizers is clearly depicted in the image. To adjust the characteristics of the molecules, the D–π–A, which is made up of a donor, acceptor, and linker section of the molecule, can be individually modified.^30^ Chains of methene units or Ar compounds, such as thiophene, can serve as conjugated linkers. Triphenylamine, indoline, perylenes, or coumarin units are some of the often-employed donors. Other than these electron donor molecules, organic sensitizers with the same electron acceptor and the π-spacer were also treated with carbazole, phenothiazine, and diphenylamine. Rhodanine-3-acetic acid and cyanoacrylic acid are examples of acceptors, which also include anchoring groups. Hydroxamate, silanol, and phosphonic acid are further anchoring groups. In addition, the stated overall efficiency ranged from 1.77% to 2.03%. The benchmarks for all dyes were also established with the inclusion of thiocyanate ligands and extra carboxylate groups serving as the anchoring sites.

Other than D–π–A configuration in DSSC, other strategies (D–D–π–A, D–A–π–A) are also employed because the push–pull effect and improved band alignment may avoid charge recombination instead of electron transfer to the semiconductor. A new method with enhanced efficiency was proposed by G. D. Carlo *et al.* in 2018 and was based on a modified porphyrin based on their Hammett constant (*σ*) in the *meso* and β (beta) positions of porphyrin. They revealed that the amine order associated with the *meso* position of porphyrin has an appreciable influence, whereas alkyl substitutions do not significantly alter the absorption spectra.^31^

Any solar technology's ability is to address an issue of energy production, which depends on striking a balance between the expenditures associated with manufacturing and installing a module (*i.e.*, a collection of functional cells), and the overall energy provided by the module, which in turn depends on the lifespan and conversion efficiency of the module. We will briefly review the significant advancements made in all areas of solar technology through research and development to provide the context for the sector. This assessment will concentrate on the successes of established first- and second-generation technologies to outline the challenges that are currently faced by the new ideas.

## Recent improvements in DSSCs

DSSC changes light energy into electrical energy dependent on the sensitization of the wide band gap of semiconductors and is principally comprises of sensitizer(dye), photocathode, electrolyte, counter terminals, and substrates glass with the transparent conductive oxide (TCO) layer. All parts of DSSCs must be improved to increase the overall efficiency.

### Substrates or conductive glass

Fine conductive glass is ordinarily utilized as a substrate because of their cheap prices, accessibility, and more optical transparency in the spectral region and close infrared districts of the electromagnetic range. Conductive covering (layer or film), as fine transparent conductive oxide, is saved on one side of the substrate. This film is considered essential since it permits sunlight to enter the cell as the directing electron bearers to the external circuit. Transparent conductive oxide materials, such as indium-doped tin oxide or fluorine-doped, are acceptable. A minimal electric charge is maintained by the conductive film for each area; there is either obstruction or rigidity. At room temperature, a common estimate of this kind of resistance is 10–20 Ω^−2^. On the conducting side, the nanostructure wide band gap oxide semiconductor (electron acceptor) is linked, fabricated, or produced.

### Photoelectrode

The nanostructure material of semiconductors is connected to a translucent leading substrate to form the photo or working electrode in a DSSC. Titanium dioxide has been the extensively used material of semiconductor material (anatase band gap 3.2 eV). Titanium dioxide is a cheap, harmless, and abundant substance. TiO_2_ nanoparticles usually comprise of 15–30 nm particle size and thickness of 10–15 μm. Screen printing and specialty blading are the most common deposition procedures for film readiness. Prior to the sintering procedure, colloidal titanium dioxide viscous glue is deposited on a substrate in one of the two approaches. Sintering is usually done between 450 and 500 °C temperatures. The rise in temperature causes electrical contact between the nanoparticles, resulting in the permeable nanostructure electrode being formed. The electrode is dye sensitized by immersing it in a dye solution for a set time. Hossein Abdi Zadeh and Mohammad Reza Golobo Stanford in their research successfully acclimatized carbon nanotubes (CNT) in the titania photoanode of the DSSC, along with various levels permeable composition made by control stage separation.^32^ The overall outcome of the experiments observed were the reduced resistance of the series, charge injection, and the longer life span of the electron. Designed for productive DSSC, the transport of charge and division properties is appealing. Furthermore, the addition of carbon nanotubes to the titania lattice increased the fundamental breadth at which the cell's optimum proficiency might be achieved. Kato *et al.* showed in their inventive methodology that the created DSSC using photoanodes made from graphene–titanium dioxide nanocomposite.^33^ The link between graphene sheet size and cell execution was explored. Stacked of cells with smaller graphene sheets were assumed to produce a larger upgrade. The adsorption of dye was increased by the smaller graphene sheets, resulting in a greater conversion productivity. Fanetal developed DSSC using photoanodes made up of two layers of nano-organized TiO_2_ sheets.^34^ DSSC efficiency based on photoanodes composite in the forms of TiO_2_ nanoparticles/nanobelts (titanium dioxide P–B), nanoparticles of titanium dioxide/nanoparticles (titanium dioxide P–P), and TiO_2_ nanobelts/nanobelts (titanium dioxide B–B) doubly stacked terminals with equal thickness of film were 3.55%, 4.81%, and 0.36%, respectively. The optical dispersion effect of nanobelts of titanium dioxide, as well as the nanoparticles of titanium dioxide and high dye consuming limit, improved the overall proficiency.

For various parameters, Saurdi *et al.* compared two monolayer photo anodes with and without ultrasonic titanium dioxide.^35^ A photoanode of titanium dioxide was used by combining the commercial titania powder P-25 with a sol–gel of titanium. The paste was subjected to ultrasonication to improve the blending state. The results showed that ultrasonication reduced monolayer molecule size, resulting in improved transmittance qualities.

The utilization of a mesoporous titanium dioxide electrode with a good inner surface area to help a sensitizer monolayer was the way to achieve the successful DSSCs in 1991. Although titanium dioxide^36^ still has the highest efficiency, many metal oxide frameworks, such as zinc oxide, SnO_2_, and Nb_2_O_5_, have also been explored. In contrast to these fundamental oxides, ternary oxides such as SrTiO_3_ and Zn_2_, SnO_4_ have also been studied, as well as center shell structures such as zinc oxide-covered SnO_2_. Huge efforts have also been made in recent years to streamline the nanostructured terminal shape, also with a huge series of nanostructures have also been tried, ranging from irregular clusters of nanoparticles to sort out arrays of nanotubes and single-crystalline nanorods. The aim for improved and coordinated charge transport along the tubes and rods as well as improved filling of the pores of entire conductor materials of the solid state DSSC inspired these investigations. The dye sensitized solar cell inner resistance affects the fill factor, conversion efficiency, and conductivity of substrates. Sangiorgi *et al.*^37^ replaced TiO_2_ sol–gel with radio frequency-sputtered titanium dioxide (RF-TiO_2_) to get greater open circuit voltage as well as shunt resistance (RSH). Titanium dioxide films with a width of roughly 100 nm were RF-sputtered at 25 °C on ITO-covered glass substrates and used as the minimized surface for DSSC fabrication. The findings showed that the RF-sputtered compact film of titanium dioxide can replace the sol–gel processing titanium dioxide compact film in the construction of DSSC when held at 25 °C. The most astonishing detailed open circuit voltage esteem for DSSCs utilizing Z-907 dye is 779 mV, which was achieved by the DSSC with an RF-sputtered compact film of titanium dioxide. Chou *et al.* presented a basic hydrolysis technique for a group manufacturing of quasi core–shell titanium dioxide (hydrolysis)/composite of lead sulphate working electrode.^38^ The assimilation spectra were found to shift from the UV to the visible range in their investigation. X. Niu *et al.* created a composite of photoelectrode with nanoparticles of platinum retained on metallic grid of a three-dimensional (3D) fluorine-doped tin oxide (FTO) as the DSSC counter terminal (CE). When compared with the standard planar counter terminal, the active surface of the iodide/triiodide redox electrolyte (I^−^/I^3−^) has increased, and the resistance of the charge transfer has decreased. The overall cell efficiency increased dramatically.^39^

According to Bekele *et al.*,^40^ various enhancements titanium dioxide time of the sensitizer adsorption was reduced from hours to minutes by quickly using the solution of the dye and the bombardments of the droplet. Abdullah *et al.* provided an antireflective compact film of titanium dioxide layer (arc titanium dioxide) including an angle refractive index that differed from the indium-doped tin oxide, altering the transmittance and reducing charge transporter loss due to recombination loss.^41^ To the creative substrate, a compact sheet at the interface provides greater right-shifted transmission pinnacles. In 2018, Seckin *et al.* introduced the molecularly-engineered Caf1 protein (in both monomeric and polymeric forms) that modified the surface states by effectively shielding unfavorable reactions and improving the light absorption properties by introducing alternative anchoring facilities. Using the novel Caf1 biopolymer with high thermal stability, they attained an unprecedented efficiency of 8.31% under standard illumination test conditions, and the output performance was maintained even under prolonged irradiation.^42^Table 1 demonstrates the compared displays of the DSSC system and made with three different kinds of photoelectrodes. The execution was compared to a solid sheet inferred by sol–gel (c titanium dioxide).

**Table 1:** Comparative efficiencies of DSSC systems made with tree main types of photoelectrodes.^43^

S. no	Kinds of photoelectrode	*J*_sc_ (mA cm^−1^)	*V*_oc_ (V)	FF	Efficiency%	Ref.
1	Nanocrystalline TiO_2_/ITO (nc-TiO_2_/ITO)	9.566	0.67	0.49	3.12	41
2	ITO/antireflective TiO_2_ compact layer (ITO/arc-TiO_2)_ (rf sputter)	11.475	0.71	0.61	5.0	41
3	ITO/carbon material – TiO_2_ (ITO/c-TiO_2_) (sol–gel)	10.63	0.72	0.61	4.68	41

The observed change in the photovoltaic and the efficiency of DSSC constructed with different photoanodes of zinc oxide was investigated. It has also been discovered that the DSSC created using arrays of zinc oxide nanowire as photoanodes can maintain more dye, boost photo usage, and offer photostimulated active sites with quick accumulation pathways, thereby improving the related proficiency. Zhu et colleagues developed a blended zinc oxide nanorod–nanosheets (NR–NS) architecture.^44^ On a zinc setting, the base created utilizing a two-step hydrothermal growth technique for DSSCs, long nanorods serve as the backbone, with thin nanosheets functioning as branches. This encouraged the photoanode for DSSC to be more adaptable. Elbohy *et al.*^45^ recently reported that adding a vanadium pentoxide (V_2_O_5_) protective sheet to the DSSC increases the productivity from 8.77% to 9.55%. In cyclic voltammetry, an increased capacitance of titanium dioxide/vanadium pentaoxide was observed as compared to uncovered titanium dioxide. The above increase in capacitance was explained as demonstrating a significant Fermi-level shift. Hydrogenation and protonation have recently emerged as unique approaches to successfully improve the electrical and photocatalytic characteristics of titanium dioxide. Su *et al.*^46^ completed a study on hydrogenated titanium dioxide (H-titanium dioxide) nanocrystals in DSSC, as previously reported. Hydrogenated titanium dioxide was arranged by strengthening titanium dioxide in an H_2_/N_2_ blended flow of the gas at temperatures ranging from 299 to 599 degrees Celsius. Compared to bare titanium dioxide, photoanodes with hydrogenated titanium dioxide nanocrystals hydrogenated at 299 °C showed the most surprising enhancement 27% in *J*_SC_ and 28% in *η* in contrast with bare titanium dioxide. Increased density of the donor, narrow band gap, and a significant shift in level band vitality of hydrogenated-titanium dioxide all contributed to the upgrade, which advances the main impetus for the infusion of electron. Acid hydrolysis, in addition to hydrogenation, is an optional technique for increasing the conductivity of DSSCs. Protons (H^+^) are deposited on the surface of titanium dioxide because of acid hydrolysis. This protonation method alters the characteristics of the surface of titanium dioxide, changes the conduction band, and improves dye–dye electrical interaction. The surface of the compact sheet of titanium dioxide was observed to have a harsh surface and additional hydroxyl bunches after being treated with sulfuric acid.^47^ The surface roughness increased the surface area, while hydroxyl clusters caused the formation of titanium oxide–titanium bonds, which strengthened the compact sheet adhesion to the mesoporous film of titanium dioxide. The effect of treatment of hydrochloric acid on the recombination of charge and edge of the conduction band growth of film of titanium dioxide was studied quantitatively by Wang and Zhou.^47^ The surface protonation of the film of the titanium dioxide resulted in a significant shift in the edge of the conduction band of 28 mV and by a factor of five slower recombination of the charge rate constant, corresponding to a 50 mV increase in open circuit voltage. The overall impact of 22 mV (50–28 mV) was consistent with the same experimentation observed open circuit voltage improvement (19 mV). The treatment of hydrochloric acid of mesoporous titanium dioxide further improves the coupling of electron between the dye's lowest unoccupied molecular orbital dimension and titanium dioxide semi-Fermi dimension, resulting in increased charge of interfacial production.^48^ Due to the elimination of recombination of the charges and a significant shift of the edge of the conduction band, the above findings demonstrated that the protonation of the surface of the titanium dioxide might improve the open circuit voltage and short circuit density at the same time.

In 2016, Seçkin Akın employed bisthiol-substituted calix^4^ arene derivatives as interface modifiers for the first time to improve the photovoltaic response of a Ru-bipy dye (N-719)-sensitized TiO_2_ photoanode in DSSCs and attained a total photon-to-electron conversion efficiency (PCE) of 12.97% *versus* a system of unmodified TiO_2_ (PCE = 6.82%) under AM 1.5G illumination of 300 W m^−2^.^49^

Finally, this section was completed with overview of literature and with the opinion that TiO_2_ is now the greatest option for semiconductors due to its low cost, abundance on the market, nontoxicity, and biocompatibility, as well as the fact that it is widely used in paints and healthcare goods as shown in Table 2.^55^ Conducting substrates such as metal foil, flexible polymer film, and conducting glass, titanium dioxide (TiO_2_) films are applied.

**Table 2:** An overview of the photovoltaic (PV) performance of dye-sensitive solar cell devices (DSSCs) along parameters using various semiconductors (SCs) as working electrodes (WEs). FF fill factor, PCE power conversion efficiency, TiO_2_ titanium dioxide, FTO fluorinated tin oxide, *V*_oc_ open-circuit voltage, *J*_sc_ short-circuit current density

Electron transport layers	PCE (%)	*J*_sc_ (mA cm^−2^)	*V*_oc_ (V)	FF	Ref.
Nanographite–TiO_2_	0.44	1.69	0.720	0.350	50
Nb_2_O_5_	3.15	6.23	0.738	0.683	51
TiO_2_ : Y_1.86_Eu_0.14_WO_6_	3.90	12.30	0.757	0.430	52
G-TiO_2_ NPs/TiO_2_ NTs	6.29	16.59	0.690	0.560	53
ONT/FTO	5.32	10.65	0.700	0.700	54

### Sensitizers

Countless efforts have been undertaken to develop various sensitizers, which can be classified into the following categories: (1) natural dyes,^56^ (2) perovskite-based sensitizer, (3) QD sensitizer, (4) metal-free organic dyes, (5) mordant dyes, and (6) Ru-complex dyes. Based on their structure, the dyes used in DSSC are divided into two main types: organic dyes and inorganic dyes. Organic dyes include both natural and synthetic dyes, whereas inorganic dyes include metal complexes such as polypyridyl complexes of ruthenium and osmium, metal porphyrin, phthalocyanine, and inorganic quantum dots. Since the confirmation of 7% based on nanocrystalline TiO_2_ by Gratzel and O'Regan in 1991,^57^ the first efficient DSSC has been based on Ru(ii)-polypyridyl dyes, and in subsequent years, the DSSCs have been based on Ru(ii)-polypyridyl dyes. However, as time went on, various concerns emerged, such as the poor absorption coefficient. To effectively catch the entire adventure light, devices must be developed with TiO_2_ films thicker than 8 mm. In this approach, modifying dye sensitizer light absorbing qualities and controlling the previously mentioned electron exchange process through basic structure design of the dye sensitizer is an important avenue for improving the DSSC efficiency. A paper on dye-sensitized fractal-type TiO_2_ electrodes with large surface areas was published in 1985 by Gratzel, Augustynski, and colleagues.^58,59^

In DSSC, sunlight capture is handled by the sensitizing dye. The sensitizer absorbs solar energy and thereby improves the cell's conductivity. A sensitizer should contain carboxyl and hydroxyl groups, which are necessary for good semiconductor binding and indicate the highest absorbance from visible to near infrared solar radiations that does not diminish quickly.^60^ Because the sensitizing dye plays such an important part in DSCC, a lot of effort has been invested into combining and testing new dyes. The ideal sensitizer should be consistent and capable of connecting to the side of the electron-directing substance.

They would be capable of retaining light at all wavelengths (*λ*) less than 923 nm, allowing them to cover the entire spectrum of light that reaches the Earth's surface while also increasing the efficacy. Because dyes are an essential component of DSSCs, there is no doubt they have captured the major interest of researchers. The three types of sensitizers are metal-complex sensitizers. In nature, metal complex sensitizers are costly, scarce, and harmful.^60^ Metal-free natural sensitizers have shown to be less effective, and the dyes' basic difficulties are their convoluted synthetic process. Natural dyes are in the form of carotenoids, anthocyanins, betalains, and chlorophyll pigments taken from blooms, natural products, plants, leaves, and roots.^61^ Natural dyes sensitized solar cells (NDSSC) have lower efficiency than metal-complex and metal-free natural sensitizers, but they use a simple extraction technique and are more environment friendly, which leads to the most recent study in the field of DSSCs.

**Fig. 7:**
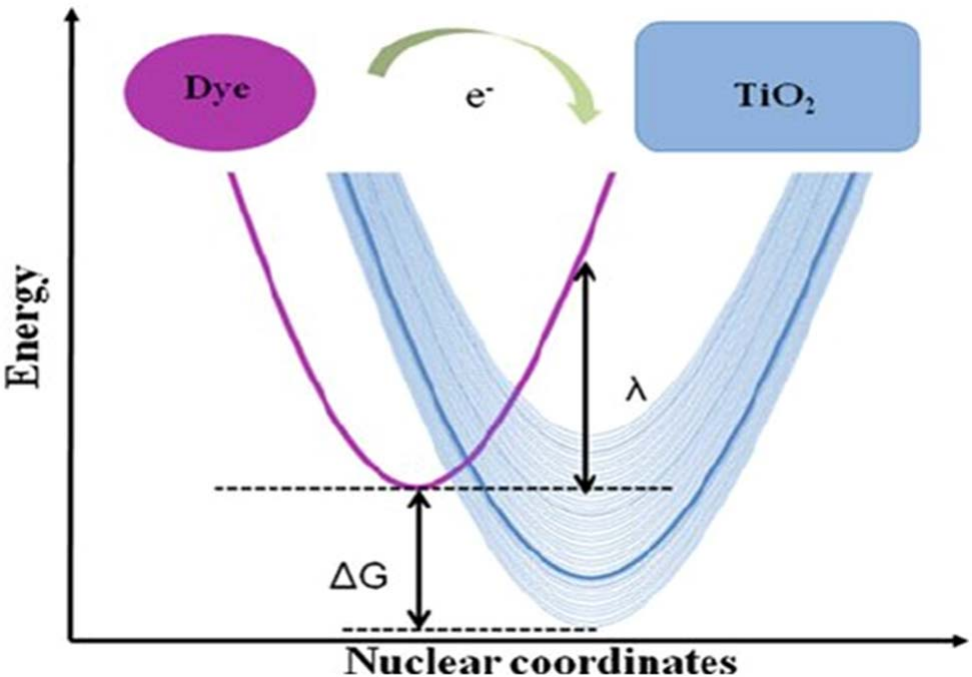
Energetics of the electron transfer process between a donor (left side) and a quasi-continuum of acceptor states (right side) typical of dye sensitization of a semiconductor.^62^

For efficient light harvesting, anchoring groups accomplished covalently bonding to –OH groups on the TIO_2_ surface and optimal absorption overlaps with that of the solar spectrum. The LUMO and HOMO energy levels must be aligned with the TiO_2_, conduction band and the iodide triiodide redox electrolyte to ensure capable electron injection and dye regeneration (Fig. 7).

#### Natural dyes

The use of ruthenium metals, which are expensive and come from typically scarce natural resources, has a significant environmental impact. As a result, using natural dyes as selective sensitizers with specific efficiencies is critical. In comparison to the expensive organic-based DSSCs, characteristic dyes provide a cost-effective choice. Various natural materials, such as flowers and vegetables, have been investigated as acceptable sensitizers in recent decades. Natural dyes are only used for educational purposes, implying that they are a less expensive and more environment friendly alternatives to ruthenium sensitizers. When the electrical structure of a pigment interacts with sunlight, the wavelengths transmitted or reflected by plant tissue are modified. Anthocyanin is a naturally-occurring phenolic molecule that gives a variety of flowers their colors, organic products (particularly berries), and vegetables. They can be found in plant tissues such as roots, tubers, and stems. The sensitizing properties of anthocyanins from different plants differ. Their aptitude to realize light and convert it into electrons has recently piqued interest of many researchers. As a result, determining the efficacy of low-cost, readily available dyes as DSSC sensitizers continues to be a research challenge. The use of natural pigments as sensitizing dyes for solar energy conversion into electricity is appealing because it improves economics while also providing significant environmental benefits. Elective sensitizers for usage with TiO_2_-based photovoltaic devices are still needed owing to the high cost of ruthenium complexes and their long-distance inaccessibility. Natural dyes present in blooms, foliage, and natural items can be extracted using simple processes under any circumstance. Characteristic dyes have long been a popular study topic due to their simplicity of use, lack of toxicity, and complete biodegradability.

In 1991, O'Regan and Gratzel reported DSSC efficiencies of 7–8%, which was an order-of-magnitude improvement. Ruthenium (Ru) dyes have gained the best achievements in recent years because of their high efficiency, upmarket cost, and complex refining.^164^ In contrast,^63,64^ as photosensitizers, DSSCs with Ru bipyridyl complexes (N3 and N719) and the black Ruthenium dye have achieved power conversion efficiencies of up to 11.2%%and 10.4%, respectively, compared to just 1% twenty years ago. The charge transfer transition by which the photoelectric charge is implanted into TiO_2_ is a significant advantage of these dyes, in addition to improved light gathering resources and staying power. In ruthenium dyes, this transfer is much faster than in the reverse reaction, where the electron recombines with the oxidized dye molecule instead of passing through the circuit and producing work. The use of hydrophobic side chains, such as that of Z-907, improves the dyes' long-term stability in solution and on the titania surface by avoiding water adsorption on the titania surface.^65^ The dye efficiency of N3, N719, K19, N945, and Z907 is 10.01%, 11.19%, 7.01%, 9.6%, and 9.61%, respectively, which is shown in Table 3. By preventing water adsorption on the titania surface, the use of hydrophobic side chains, such as those found in Z-907, improves the dyes' long-term stability in the solution and on the titania surface.

**Table 3:** The photoelectrochemical properties of dye sensitizers based on ruthenium

Dye	*J*_sc_ mA cm^−2^	*V*_oc_	FF	*η* (%)	Ref.
N3	18.21	720	0.730	10.01	66
N719	17.74	846	0.750	11.19	67
Black dye	20.91	736	0.722	11.11	65
Z907	14.60	722	0.693	7.30	66
K19	14.62	711	0.671	7.01	68
N945	16.52	790	0.720	9.61	69

The power conversion efficacy of organic dye-sensitized DSSCs is slightly lower than that of metal complex-sensitized DSSCs. Pure organic dyes, on the other hand, have several advantages for DSSC, including a lower cost, higher absorption coefficient, and more accessible redox potential force. To get even cheaper dyes, metal-free organic dyes are strongly desired for DSSC.

Wongcharee and his associates have used a few natural dyes so far as sensitizers in DSSC.^70^ Only certain pigments are suitable for converting sunlight into electricity, according to the DSSCs, which contained various pigments with varying photosensitizing effects. Because of the greater interaction between betalain and TiO_2_, the betalain pigment has the finest execution.

Chang *et al.* investigated various operating parameters utilizing chlorophyll dye from anthocyanin and pomegranate coloring from mulberry soil materials. A maximum efficiency of 0.722% was seen when chlorophyll and anthocyanin were combined. The DSSC was effective at 0.597% and 0.548%, respectively, when pomegranate leaves and mulberry organic product were used.^71^ Narayan *et al.* used *Allamanda cathartica*, *Bougainvillea spectabilis*, and *Cosmos sulphureus* to create the DSSC. Maximum absorbance is observed at 383 and 542 nm wavelengths in the presence of bougainvillaea. Open circuit voltage, current density, fill factor, and productivity were 0.359 V, 0.898 mA cm^−2^, 0.52%, and 0.38%, respectively, as shown in Table 4. Individually, the efficacy of using brilliant trumpet and Cosmos as a distinctive dye was 0.40 and 0.54, respectively.^72^

**Table 4:** Different natural dyes with photovoltaic parameter.^72^

Dye	*J*_sc_ (mA cm^−2^)	*V*_oc_ (V)	FF	Efficiency (%)
Bougainvillea	0.878	0.359	0.52	0.38
Golden trumpet	O.878	0.405	0.54	0.40
Cosmos	1.041	0.447	0.52	0.54

Hernandez-Martinez *et al.* enquired about violet *Bougainvillea glabra*, red *Bougainvillea glabra*, red *Bougainvillea spectabilis*, and cleaned red *Bougainvillea glabra*. The most noticeable execution trademark is red *Bougainvillea spectabilis*, which has a 0.48% efficacy.^73^ McHale *et al.* used liquid chromatography to isolate betanin from beet root at a medium pressure and discovered a 2.7% increase in cell productivity. On the other hand, anthocyanin and chlorophyll dyes demonstrated decreased efficacy when extracted transparently, betanin pigments demonstrated a significant increase in the cell proficiency when extracted differently. Although a lower open circuit voltage of DSSC was observed, this value should be increased further.^74^

Magaraphan *et al.* investigated the functional characteristics of two unique DSSCs using pure titania and titania-clay as the semiconductor sensitized with natural sensitizers such as blue pea, rosella, and red cabbage. The red cabbage with pure TiO_2_ semiconductor displayed an astonishing 0.13% efficacy. Regardless, less proficiency was recorded.^75^ Jasim *et al.* examined the optical and electrical properties of DSSCs utilizing colors taken from henna from Bahrain, raspberries, grapes, and pomegranate. The absorption of henna dye from Bahrain was greatest in the near-infrared visible, ultraviolet, and areas of the solar spectrum. In addition, the pomegranate and Bahraini raspberries demonstrated comparative responsiveness (Table 5).^76^

**Table 5:** Summary of parameters of the DSSCs with various dye sensitizers.^76^

Sensitizer	Fill factor (FF)	Current density (mA cm^−2^)	Open circuit voltage (*V*_oc_)	Efficiency (*η*) %
Yemeni henna (87 g)	28.1	0.407	0.306	0.117
Bahraini henna (80 g)	24.6	0.368	0.426	0.128
Pomegranate juice (100 g)	48.1	1.700	0.395	1.076
Raspberries (32 g)	45.5	0.566	0.360	0.309
Cherries (97 g) + 1% HCL	28.8	0.463	0.301	0.134
Cherries (97 g)	38.3	0.466	0.305	0.181

C. I. Oprea *et al.* investigated both the practical and theoretical components of betalain organic dye. The density functional theory computations are illustrative of the various electronic characteristics of betalain, while the exploratory results indicated that distillation increased the short circuit current and efficacy.^77^ T. M. El-Agez *et al.* produced a DSSC by sensitizing it using and sumac/rhus leaves and *Lawsonia inermis* products from the *Curcuma longa* root. The solid-state thin film electrolyte is composed of 50% chitosan and 50% polyethylene oxide. To obtain the I/I^3−^ redox pair, the polymer mix complex with ammonium iodide (NH_4_I) and few iodine crystals were combined with the polymer NH_4_I solution. At ambient temperature, the polymer electrolyte had an ionic conductivity of 1.18105 S cm^−1^. With red purple sumac/rhus extract, the maximum proficiency, current density, open circuit voltage, and fill factor were 1.5%, 0.93 mA cm^−2^, 394 mV, and 0.41, respectively (refer to Table 6).^78^

**Table 6:** DSSC performance with natural sensitizer.^78^

Natural sensitizer	Current density (mA cm^−2^)	Open circuit voltage (*V*_oc_)	Fill factor (FF)	Efficiency (*η*) %
*Lawsonia inermis* leaves	0.38	336	0.57	0.7
Sumac/Rhus fruits	0.93	394	0.41	1.5
*Curcuma longa* roots	0.20	280	0.65	0.36

Taya and his group utilized the seeds of 3 different plants, namely, *Dianthus barbatus*, *Lepidium sativum*, and *Raphanus raphanistrum*, as natural dyes. *Dianthus barbatus* has the greatest reported fill factor and efficacy at 0.48 and 0.15, respectively. Both *Lepidium sativum* and *Raphanus raphanistrum* seeds exhibited a fill factor of 0.45 and a proficiency of 0.5%.^79^ Latif *et al.* produced dye-sensitized cells with eleven different colors sourced from diverse tree species. The sensitizer was obtained from the flowers, leaves, bark, and underlying structures of three species. The DSSC that utilized zizyphus leaves as a dye had the greatest success rate of 0.40% among all the sources.^80^ Alwani *et al.* synthesized natural dyes from *Cordyline fruticosa*, *Pandanus amaryllis folius*, and *Hylocereus polyrhizus* by dissolving them in nine dissolvable solvents: n-ethanol, hexane, chloroform, acetonitrile, oil ether, ethyl-ether, and *n*-butyl liquor. Among all, the polyrhizus dye exhibited the highest adsorption.^81^ Kumara *et al.* created a unique dye formulation with shisonin and chlorophyll and attained the highest transformation yield of 1.31%.^82^

Olea *et al.* investigated how black berry concentrate can be used to light-sensitize TiO_2_.^83^ When black berry extracts sensitized TiO_2_, the photocurrent reaction increased, indicating an abundance of photoelectrons due to the concentrate's light absorption. The short current density (*J*_SC_), open circuit voltage (*V*_OC_), and proficiency (*η*) of DSSCs were determined to be 4 mA, 300 mV, and 1%, respectively, for a 4 cm^2^ dynamic region cell. Garcia *et al.* were used the fresh extracts of chaste tree fruit, mulberry, and cabbage-palm tree fruit as TiO_2_ sensitizers in thin-layer sandwich-type photoelectrochemical solar-powered cells.^84^ Natural sensitizers were used to develop visible light to power conversion, yielding *I*_sc_ and *V*_oc_ values that were comparable to orchestrated dyes. According to Hao *et al.*, the short circuit current (*I*_sc_) measured from 0.5 cm^2^ of DSSCs using fresh extracts of modest tree organic product, mulberry, and cabbage-palm tree fruit as sensitizers was 1.06, 0.86, and 0.37 mA, respectively.^85^ The maximum power (*P*_max_) ranged from 58 W to 327 W, with an open-circuit voltage (*V*_oc_) ranging from 0.551 V to 0.412 V, a fill factor of 0.52 to 0.63, and the short circuit current (*I*_sc_) varied between 1.142 and 0.225 mA. Because of the improved cooperation between the carbonyl and hydroxyl gatherings of anthocyanin atoms on dark rice separate and the surface of TiO_2_ permeable film, black rice extracts had the best photosensitized influence in the concentrates of natural fruit, leaves, and flowers. Fernando and his companions considered dyes extracted from tropical flowers as potential TiO_2_ sensitizers by accumulating DSSC.^86^ Photovoltages range from 390 to 410 mV with photocurrent densities ranging from 1.1 to 5.4 mA cm^−2^. The overall efficacy and fill factor of these cells ranged from 0.2 to 1.1 and 0.53 to 0.64, respectively.

Table 7 shows the status of DSSC photoelectrochemical^90,91^ parameters that are dependent on natural dyes. The proximity of carboxylic acids in the betalains provides an advantage for anchoring, as does the increased oxidation potential. The betalain hues in red turnip are concentrated^89^ and was the second most-effective. The productivity of DSSC sensitized with pomegranate juice was 1.50%. At its most basic level, pomegranate juice includes cyanin subordinates and exists as flavylium at a specific pH. Natural dyes were extracted from natural resources such as blooms, leaves, natural goods, traditional Chinese medicines, and beverages and used as sensitizers in the DSSC manufacturing process. In the photoelectron chemical execution of the DSSC based on these dyes, the *V*_oc_ ranged from 0.38 to 0.69 V and *J*_sc_ from 0.14 to 2.69 mA cm^−2^. Moreover, Roy *et al.* demonstrated that their DSSC's *J*_sc_ and *V*_oc_ reached 3.22 mA cm^−2^ and 0.89 V, respectively, while using Rose Bengal dye as a sensitizer, it yielded a 2.09% change efficiency.^92^ A dye-sensitized solar cell was produced by Huizhi Zhou *et al.* employing a natural dye as a sensitizer. The organic color was created with *Rosa xanthina*, *Capsicum*, *Erythrina*, black rice, kelp, and variegata bloom. Among all dyes, blue rice extracts and produces the greatest results. The *V*_oc_, *J*_sc_, fill factor (FF), and intensity of DSSC were determined to be 551 mV, 1.142 mA cm^−2^, and 0.52, respectively.^93^ The natural color sensitizer cyanine (flavylium) was isolated from organic pomegranate products by Sirimanne *et al.* solid-state TiO_2_/impregnated natural dye, *i.e.*, pomegranate piment/CuI (*p* type) solar device showed the *λ*_max_ at 570 nm, which indicates the cell's highest efficiency when compared to other natural dyes (santalin, cyanidin, nutrient C, and tannin).^94^ Senadeera *et al.* isolated a variety of distinctive dyes from tropical blossoms (*Sesbania grandiflora* red, *Rhododendron arboretum zeylanicum*, *Nerium oleander*, *Hibiscus rosainensis*, and *Ixora macrothyrsa*, *Hibiscus surattensis*).^86^ The overall efficacy increased from 0.2 to 1.1%, while the current density increased from 1.1 to 5.4 mA cm^−1^. Hibiscus and its counterparts exhibited the highest proficiency rate of 1.14%.^86^*Cannabis indica* L, cowberry, *Salvia splendens*, and *Solanum nigrum* L were found to contain a naturally-occurring sensitizer by Luo *et al.* with a high resistance (27 902 Ω) to cowberry dye at the TiO_2_/dye/electrolyte contact, indicating that the cell did not work well at all. In contrast, *Cannabis indica* has the highest cell proficiency of 0.29% because of its decreased interfacial resistance.^95^

**Table 7:** The photoelectrochemical parameters of certain natural dye-based DSSCs

Dye	*J*_sc_ (mA cm^−2^)	*V*_oc_ (V)	FF	*η* (%)	Ref.
Festucaovina	1.188	0.550	0.696	0.47	87
Tageteserecta	2.892	0.478	0.609	0.99	88
Rosella	1.68	0.45	0.56	0.39	89
Black rice	1.15	0.55	0.55	—	85
Pomegranate	0.20	0.40	0.45	1.50	89

Chang *et al.* decolorized spinach and ipomoea extracts. Spinach and ipomoea were found to have the highest absorbance at wavelengths of 437 nm and 410 nm, respectively. Ipomoea alone achieved the highest efficiency or productivity of 0.278%. In addition, it has been demonstrated that an increase in temperature has a remarkable effect on the execution of DSSC.^71^

Muthukumarasamy *et al.* evaluated the unique properties of natural dyes derived from *Eugenia jambolana* and *Delonix regia* using two separate electrolytes (liquid and quasi solid polymer). Eugenia with his group found a maximum effectiveness of 0.5% when used in conjunction with a liquid electrolyte. This effectiveness was insufficient for commercialization.^96^ Thambidurai *et al.* synthesized DSSC sensitized with natural concentrates of mulberry, *Ixora coccinea*, and beetroot. Among them, the mulberry separation revealed the most astonishing effectiveness. Individually, the proficiency with mulberry, *Ixora coccinea*, and beetroot was 0.41%, 0.33%, and 0.28%, respectively.^97^

Kim *et al.* organized and analyzed natural dye collected from the blooms of *Kerria japonica*/*Rosa chinses* with and without sugar molecules. The proficiency of the carotenoid dye derived from *Kerria japonica* grew from 0.22 to 0.29% as the sugar concentration increased, while small decreases were observed if *Rosa chinensis* extract included anthocyanin.^98^

#### Metal-free organic dyes

Hemicyanine, merocyanine, squarylium, and cyanine dye have shown adjustable absorption in the red to near infrared region and high absorptivity, making them promising sensitizers for DSSCs. Due to strong intermolecular van der Waals forces, it has been discovered that a portion of these dyes have a strong tendency to self-associate in arrangement or at the solid–liquid interface.^99,100^ The aggregates retain the dye differently than the monomeric species, and three different collecting examples of the dye have been proposed: red-shifted J aggregates and combined blue- and red shifted herringbone aggregates.

Notable progress has recently been produced in the realm of deeply engrossing metal-free organic dyes with the most noteworthy solar directed to electric power transformation proficiency increasing by 9%.^101,102^ This is accomplished by the development of appropriate molecule structures, their tunable absorption, and desirable electrochemical characteristics. These metal-free organic dyes have a clear lead.^103^

To obtain large IPCEs in DSSCs, it was determined that a widening of the intake *via* controlled accumulation is required.^59,104,105^ However, because of the aggregate configuration during adsorption on the TiO_2_ surface, these dyes exhibited poor power transformation efficiency in solar cells. Similarly, one of the major breakdown mechanisms for these dyes is *cis*–*trans* photoisomerization.^106^

Metal-free organic dyes are less expensive and have better molecular structure differentiation and molar extinction coefficients. Novel photosensitizers based on coumarin, cyanine, indoline, triphenylamine, hemi cyanine, merocyanine, dialkyl aniline, tetrahydroquinoline, phenothiazine, and carbazole have recently achieved solar-to-electrical power conversion efficiencies of up to 9%.

The Li group investigated new cyanine trimethyl cyanine derivatives, pentamethyl cyanine derivatives, a mixture as photosensitizers.^107^ DSSC had the highest photoelectric conversion of 3.4% based on their combinations. A photoelectrochemical cell containing flavonoid anthocyanin dyes extracted from dark berries could convert daylight to electrical power with a competence of 0.56% under full sun, according to Cherepy *et al.*^108^ Open-circuit voltages of 0.4–0.5 V and short-circuit photocurrents of 1.5–2.2 mA cm^−2^ were attracted to such a simple structure, which prompted proficient charge transporter injection. The amounts of flavonoid glycosides produced by the two concentrations differed.^86^

#### Metal-complex photosensitizer

Zhu *et al.* announced two new sensitizers dependent on triphenylamine-dicyano vinylene and utilized for the p-type dye-sensitized solar cells.^109^ This investigation proposed that the change in the crossing over the moiety among the triphenylamine and the carboxylic gathering by cumulative thiophene units is a promising path for turning away charge recombination; thus, the control conversion efficiency can be supported. The two dyes were coded T3 and T4. Aside from the amalgamation of novel dye, different systems have been utilized to enhance the exhibitions of the DSSC utilizing the existing dye. A work done by Li *et al.* reported two new cyclometalated ruthenium sensitizers NC102 and NC103, where the two NCS-ligands of the N3 simple supplanted with the 2-thiophen-2-yl-pyridine and 2-benzo[*b*]thiophen-2-ylpyridine ligands, respectively, were synthesized for DSSC applications.^110^ The amalgamation of thienyl pyridine ligands in the ruthenium complexes improved the other worldly reaction and red-shifted retention maxima in the noticeable area. The charge transfer resistance was observed to be high in every one of these dyes. The electron lifetime was observed to be shorter, which can be induced from the slighter short circuit photocurrent and lower by and large productivity change of each DSSC. Zhai and associates got an overall efficiency of 3.4% utilizing quinque thiophene dicarboxylic acid as the sensitizer.^111^ In another examination work, Y. Tachibana *et al.* incorporated two new D–A–pi–An indoline dye (X S 45 and X S 46) with various extra donor to examine the impact of donor and bridge structure in indoline dye on the photovoltaic properties of DSSCs utilizing iodine/cobalt electrolyte.^112^ The massive dipropylfluorene unit presented in the donor part essentially upgraded the light energy ingestion capacity and remarkably delays the charges recombined particles at the titania/electrolyte interface. The presentation of the benzothiadiazole (BTD) in the spacer evinces a hefty receptive range of wavelengths into the NIR region, yet additionally diminishes the molar absorption coefficients of the indoline dye.

To retain distinct areas of the UV visible spectrum, Y. Chen *et al.* combined a yellow merocyanine dye (max at 380 nm), a red hemicyanine dye (max at 535 nm), and a blue squarylium cyanine dye (max at 642 nm). The absorption range of the co-sensitized devices was 350 to 750 nm, resulting in a general efficiency of 6.5%, which was higher than that of single-sensitizer reference devices. These three dyes were refined together, which reduced the overall aggregation and changed the photocurrent and infusion efficiencies.^113^ Using fluorescent perylene, liquid electrolytes were created and coupled in DSSC by Shibano Y., *et al.*^114^ Due to the spectacular down-moving property of perylene, photons with short wavelengths (between 350 and 440 nm) can be digested and then transformed by more than 10 to ones with longer wavelengths (between 450 and 550 nm), which can be more productively used by DSSCs. As a result, the device with optimal centralization of 0.05 M perylene exhibits a viable change in the short out current thickness (*J*_sc_), resulting in an 11.6% increase in power transformation productivity (PCE) when compared to the reference DSSC in the presence of the control electrolyte.

It was discovered by K. Sayama *et al.* that when the length of the alkyl side chain attached to the benzothiazole ring increased and the methylene units between carboxylic acid group and dye chromophore reduced, the conversion efficiency and IPCE value improved as they investigated a series of benzothiazole merocyanines with varying alkyl chain lengths.^59,115^ It had a *J*_SC_ value of 11.4 mA cm^−1 2^ (4.5%) and the longest alkyl chain (*n* = 18; *p* = 1; *m* = 1) was the most effective sensitizer in terms of efficiency. It was found that the length of the excited dye affected the rate at which electrons were transferred to the TiO_2_ conduction band by increasing the amount of C–C two-fold securities used in the dye synthesis.^115^

Following the photoanode, the dye or sensitizer plays a crucial function in capturing the sun's light energy. To ensure the viability of the retention technique, the light-capturing capability of the dye in both the near-infrared and visible areas must be remarkable. In the growth of unique dyes, several efforts have been made to integrate new dyes that may boost the DSSC's overall efficacy. Numerous researchers have advanced their research using new synthesizers, with novel synthetic and physical features further need to be discovered. A few of those that displayed exceptional input have been highlighted here. Sufficient effort has been expended over the years to improve the dye in every way possible.

Liu *et al.* described the meticulous planning of four artificial chlorine-type sensitizers. The high rate of chlorin absorption in the Q band areas contributed to improving the overall solar efficiency of DSSC.^116^ The light energy transformation efficiency of the DSSC is intrinsically tied to the molecular structure of the grafted dye. For example, if a molecule has an anchoring group at 20 positions, adding a 2,6-dichlorophenyl group to either the (5) or (15) position increases the efficiency of photon energy to electricity conversion. Zhang *et al.* accounted for the structure of three dyes using a variety of electron donors, including carbazole, indoline, and coumarin.^117^ The work investigated two critical components of the sensitizer that influenced the *V*_OC_. The first part is charge recombination, which occurs when the electrolyte and dye combine. The other is the sensitized adsorption-induced TiO_2_ conduction band energy shift (DECB). Mao *et al.* isolated three comprehend organic sensitizers from the C219 precursor and used only unique electron donors to increase the cell's *V*_OC_.^118^ Wu *et al.* projected the synthesis of two new organic dyes called the mas J5 and J6 that contain julolidine as the donor group (D) and cyano acidic corrosive or rhodanine-3-acidic corrosive as the electron acceptor (A) coupled *via* a bithiophene unit. The results confirmed that sensitizers are rapidly recovered and that the redox arbiter successfully captures the dye cations.^119^ The recombination rate was significantly increased by substituting rhodanine-3-acitic acid for cyanoacetic acid.

According to a study published by Li *et al.*, two new cyclometalated ruthenium sensitizers NC102–103 were synthesized for DSSC applications.^110^ The two NCS-ligands of the N3 simple were replaced with 2-thiophen-2-yl-pyridine and 2-benzo[*b*]thiophen-2-ylpyridine ligands, respectively. The modification of ruthenium complexes by the incorporation thienyl pyridine ligands enhanced the extraterrestrial reaction and bathochromically shifted the retention maximum in the detectable range. Each of these dyes exhibited a strong charge transfer resistance. The lower electron lifespan can be attributed to the weaker short-circuit current and the decreased productivity of each DSSC. Reddy *et al.* orchestrated the development of four novel organic dyes designated CSORG6, CSORG7, CSORG8, and CSORG9.^120^ The dyes used electron-dense thiophene spinoffs as the donor moiety and cyanoacrylic corrosive as an acceptor, with phenothiazine or phenoxazine acting as a crossover agent. The alkyl-substituted thiophene units may boost the dyes' light gathering potential. Apart from the incorporation of unique dyes, several approaches have been used to enhance the DSSC's presentations using current dyes. Mozaffari and his coworkers produced polyepinephrine (PEP) and polydopamine (PDA) in an basic medium comprising tris(hydroxymethyl)amino methane (THAM) buffer with a pH of 14 and 8.5 in a nitrogen atmosphere.^121^ The excessive hydroxyl OH functional moiety acts as an electron donor and allows the polymer to develop, increasing the length chain of the polymeric fiber. The HOMO and LUMO energy difference decreases as the polymer's length grows and a reduced band gap is achieved. In addition, the polymer's solubility in solution reduces and particles are spread in the solution, resulting in an essential bathochromic shift of the absorption peak. This results in increased short circuit currents and conversion efficiency. In another study conducted by Lee *et al.*, cyanoacrylic acid was combined with phenoxazine to facilitate electron transfer from the D moiety, and an *N*-substituent was incorporated to inhibit dye buildup.^122^ Fang *et al.* advocate expanding the dye's adsorption and desorption cycles to ensure proper dye particle conveyance and, subsequently, increased the photoconversion productivity.^123^Table 8 summarizes the photovoltaic performance of various synthesized dyes.

**Table 8:** Summary of photovoltaic parameters of various dyes employed in DSSCs

S. no.	Novel dyes	Type	*V*_oc_ (V)	Efficiency (%)	*J*_sc_ (mA cm^−2^)	FF	Ref.
1	D–A–pi–A indoline dyes	Type XS45	0.686	6.90	14.8	0.68	116
		XS46	0.640	5.87	13.3	0.69	
2	Cyclometalated ruthenium sensitizers	Type NC102	0.63	3.64	8.15	0.71	110
		NC103	0.63	4.22	9.45	0.71	
3	New organic dyes	Type CSORG6	0.645	4.3	9.19	0.725	120
		CSORG7	0.726	6.0	11.56	0.719	
		CSORG8	0.694	5.4	10.84	0.724	
		CSORG9	0.720	6.0	12.07	0.685	
4	New dyes for P-type DSSC	Type T3	4.01	0.144	0.33	0.19	124
		T4	1.69	0.123	0.29	0.06	

Kumara *et al.* dyed a ss-DSSC with p-CuI as the gap conductor using chlorophyll, shisonin, and as the blender of the two-dye derived from shiso plants. The highest productivity increase achieved with a combination drink dye (shisonin and chlorophyll) was 1.31%. Individual DSSCs based on shisonin and chlorophyll demonstrated proficiency of 1.01% and 0.59%, respectively. The open circuit voltage (*V*_oc_), current density (mA cm^−2^), FF, and efficacy (percentage) when shisonin is used are 550, 0.59, 0.51, and 1.01, whereas when chlorophyll is used, they are 432, 3.52, 0.39, and 0.59, respectively. In addition, the mixed dye solar cell achieved the highest efficiency of 1.31%.^82^ In 2012, using a Fe^2+/^Fe^3+^ (ferrocene) liquid electrolyte and natural dyes extracted from *Hypericum perforatum*, a novel and promising DSSC bilayer design was developed. A quercetin-based DSSC demonstrated the highest solar-to-electricity conversion efficiency compared to other dyes, with a maximal value of 2.17%.^125^ Yamazaki *et al.* investigated the natural sensitizers crocin, carotenoid, and crocetin. It was discovered that crocetin- and carotenoid-containing carboxylic (–COOH) groups may adsorb viably on the semiconducting material, elevating in the cell's greatest performance, while crocin demonstrated decreased productivity due to the absence of the carboxylic group.^126^ Furukawa *et al.* developed two distinct dye solar cells by incorporating varied molecular weight polythene glycol (PEG) into the oxide paste. Red cabbage and curcumin were used to modify the color. The productivity of cells containing PEG with a molecular weight of 2 000 000 was found to be high (0.99%) when compared to cells containing PEG with a molecular weight load of 50 000 (0.42%).^127^ Calogero *et al.* developed NDSSCs by sensitizing them with bougainvillaea blooms, purple wild, red turnip, and Sicilian thorny pear natural product squeeze. On comparing betalain hues with the N719 ruthenium complex dye, the efficiency of natural concentrates was found to be smaller (1.26%).^128^ Taya *et al.* discovered a dye that may be made from fresh or dried basic materials. Sensitizers were made from the leaves of five separate plants. The largest discovery was made regarding spinach oleracea extract. Two distinct DSSCs were collected utilizing nanostructured mesoporous TiO_2_ and ZnO films, the outcomes are summarized in Table 9,^129^ and the conductivity parameters of the DSSC employing the various distinctive dyes are summarized.

**Table 9:** Summary of photovoltaic parameters based on ZnO and TiO_2_ electrodes for DSSC^129^

Electrode	*J*_sc_ (mA cm^−2^)	*V*_oc_ (V)	*J*_m_ (mA cm^−2^)	*V*_m_ (V)	*P*_m_ (mW)	FF (%)
ZnO	0.123	0.226	0.065	0.101	0.008	20
TiO_2_	1.11	0.583	0.775	0.387	0.301	46

The addition of graphene to the dye boosts the performance of DSSCs. Chen *et al.* coated the working electrode with graphene at room temperature, rendering it permeable and thus enhancing dye adsorption. The efficiency of the cell rose from 5.98% to 6.86%.^130^ Enriquez *et al.* boosted the production of natural dyes by mixing graphene, anthocyanin dye, and titanium dioxide from red cabbage. The effectiveness of natural dye coated with graphene was 0.51%.^131^

### Electrolytes

Electrolyte is critical in the DSSC as the electrolyte enhances the charge transport between the counter anode and the photoelectrode. Researchers from all around the world have done substantial study on the I^−^/I^3−^redox pair as an electrolyte for DSSCs. For solid-state DSSC applications, I^−^/I^3−^ incorporated into a polyaniline/thiourea matrix is also employed. Due to this optimization, the short-circuit current density and open-circuit voltage have increased, which significantly increased the PCE.^132^ The basic function of the electrolyte is to recover the dye at the regeneration step. The electrolyte should be light, with a low vapor weight, high boiling point, and good dielectric properties.^133^ Low viscosity, low vapor pressure, high boiling point, and outstanding dielectric properties characterize the perfect electrolyte solvent. Industrial factors such as robustness (compound latency), natural regeneracy, and ease of preparation are also crucial. The most often utilized electrolytes are I^−^/I^3−^ inorganic solvents, inorganic ionic fluids, and solid electrolytes. The best redox mediator currently employed in DSSC is a liquid electrolyte that holds the redox pair. Ionic liquids containing iodide/triiodide have qualities such as substance stability and exceptional ionic conductivity, making them a possible alternative electrolyte. Finding a common redox pair will be one of the most difficult tasks for future DSSC experiments along long-term stability. When an inorganic ionic electrolyte is present, the efficiency of the system degrades over time. The leakage-free property of the strong electrolyte sets it apart from all other electrolytes.^134^ The best p-type strong material with a large band gap that is simultaneously transparent and affordable is copper iodide,^135^ although it has many shortcomings and problems to be resolved.

Electrolytes for DSSCs are classified into three types: liquid electrolytes, solid state electrolytes, and quasi solid-state electrolytes.

#### Liquid electrolytes

Liquid electrolytes are basically classified into two types: organic solvent-based electrolytes and room temperature ionic liquid electrolytes (RTIL) based on the solvent used.

#### Ionic liquids as an electrolyte

Ionic liquids (ILs) are new DSSC solvents, liquid parts, and quasi solid electrolytes.^136,137^ The key advantages of ILs over organic solvents are their decreased instability (owing to low vapor pressure), strong ionic conductivity, and thermal soundness, leading to solar cells with greater long-pull resilience. Devices with liquid electrolytes will generally decay faster than cells with ILs in terms of performance. Electrochemical dependability is usually desired when ILs are employed instead of liquid electrolytes. Numerous additional ionic liquids have also been researched for their possible use as solvent-free electrolytes in DSSC, including ammonium,^138^ guanidinium,^139,140^ phosphonium,^141,142^ pyridinium, and sulfonium.^143–145^ However, due to their high viscosity and issues with mass-transport, these have not produced good efficiency.^142,145^ It is interesting to note that improved IL concentrations, such as imidazolium iodides, can also help to effectively reduce the dye molecule, which improves the DSSC performance.^146^ Very outstanding (>10%) efficiency under full sun illumination has been successfully demonstrated by mixing organic solvents with optimum concentrations of ILs.^147^ Ionic liquids (ILs) maintain high melting points and high viscosities. The key benefit of an IL-based electrolyte is the absence of the risk of leakage from the cell channel, which compromises the DSSCs' long-term operational stability. The first stable DSSC was demonstrated using an IL-based electrolyte that contained methylhexyl-imidazolium iodide (MHImI), and it did not exhibit any performance decline.^148^

ILs are further classified as follows.

RTILs (room temperature ionic liquids) maintain low viscosity and a lower melting point (<100).^149,150^ They are a class of organic salts made up of anions from the halide or pseudohalide family and captions such as pyridinium and imidazolium (Table 10).^151^

**Table 10:** The highest recorded efficiency using ionic liquids

Electrolyte	Dye	Long term stability	PCE (%)	Ref.
DMII, I_2_, NBB GuNCS, NaI in BN	C106	1000 h at 60 °C	10.0	142
I_2_, NMBI in PMImI/EMImTCM	Z907Na	672 h at 60 °C	7.40	143
I_2_, GuSCN, TBP in PMImI/EMImSCN	Z907	1000 h at 55–60 °C	7.0	152
I_2_, 0.5 M NMBI, 0.1 M GuSCN in PMImI/EMImB(CN)_4_	Z907Na	1000 h at 60 °C	7.0	153
PMII, 4-OH-TEMPO, NOBF_4_, LiTFSI, NBB in MPN	D205	800 h at 25 °C	7.20	154

#### Solid-state electrolyte

The liquid electrolyte that has historically been used in DSSCs has several drawbacks, including poor long-term constancy due to fluid combustibility, spillage, dye breakdown, and leakage, can significantly reduce the solar cells' long-term stability. Solid state electrolytes have been developed to improve the performance and stability. They substitute a p-type semiconductor for the liquid electrolyte.^155^ Because they have ionic flexibility required for effective connections between segments inside the solar cell, polymer-based gel electrolytes can be used as a solution. Although several quasi solid-polymer electrolytes have been used in DSSCs, the transformation efficiencies obtained are often low when associated to those obtained with liquid electrolytes.^156–158^ Amalina *et al.* investigated the effect of copper(i) iodide (CuI) solution concentration on the thin film's characteristics and photovoltaic performance.^159^ CuI thin films deposited on glass substrates were studied for surface morphology and electrical properties. The results revealed that the precursor concentration has a significant impact on the CuI thin film characteristics. This suggests that fog atomization could eventually replace the current hole transport material deposition innovation in the commercial manufacturing of solid-state DSSC. With inorganic nanofillers (TiO_2_, SiO_2_, Al_2_O_3_) and polyethylene glycol, M. Amalina and M. Rusop^159^ developed efficient composite electrolytes (PEG). According to morphological and physical analysis, the crystallinity of the Al_2_O_3_–PEG composite electrolyte was lower than TiO_2_–PEG, SiO_2_–PEG electrolyte, and PEG.

#### Counter electrode

A DSSC's counter electrode is another essential component. The decrease in triiodide is the primary motive for the counter electrode. The lower reaction rate at the cathode is crucial because triiodide is reduced to iodide, which is then used to recover the oxidized dye particle at the anodic side of the cell. An achievable cell requires a modest response on the anodic side and a rapid response on the cathodic side, which is the counter terminal. The counter terminal is near to the redox couple's equilibrium capacity, but the anodic side is a long way from the equilibrium potential. This phenomenon causes a voltage contrast in the DSSC, resulting in a drop in the triiodide levels. Although a variety of materials such as carbon, platinum, conductive polymers, and graphite are used as counter anodes, platinum remains the preferred catalyst. For the iodide/triiodide redox pair, platinum is a superior catalyst. Furthermore, the light reflection coefficient of platinum is larger than that of carbon, causing all the light to enter the cell. The electrolyte is recovered by the counter cathode. Platinum is the best material as a counter cathode, resulting in great cell productivity even though it is an expensive material. Carbon, on the other hand, is a less expensive and adequate reactant.^128,160^ Ahmad *et al.*^161^ presented counter cathodes made from multiwalled carbon nanotubes (MWCNT) and graphene nanoparticles (GNP). Materials are isolated using PEDOT:PSS polymer and then placed using the drop casting technique on FTO glass and nonconducting glass substrates. The counter anode's life and effectiveness have been improved in his work. According to Y.-H. Lai *et al.*,^156^ an approach for producing profoundly transparent platinum counter anodes founded on the spray coating of Pt nanoparticles on heated substrates has been developed. As a result of the 86% reduction in platinum usage, the fabrication cost was lowered. A nanostructure-based Pt counter terminal was developed by gathering silver nanoparticles on glass substrate and keeping a thin layer of Pt in another effort by M. Wang *et al.* to improve the effectiveness of the DSSC.^162^ DSSCs with nanoparticulate structures have improved the photon-to-current transformation efficiency, power conversion efficiency, and short-circuit current. Table 11 shows the DSSC performance parameters after counter electrode modification.

**Table 11:** DSSC performance parameters after counter electrode modification

S. no	Counter electrode proposed idea	Type of CE	*J*_sc_ (mA cm^−2^)	*V*_oc_ (V)	FF	Efficiency (%)	Ref.
1	Counter electrodes using nanoparticles	Hybrid/glass type CE	8.274	0.66	0.48	2.47	161
		PEDOT:PSS/glass type CE	0.683	0.46	0.43	0.14	
		GNPs/glass type CE	2.43	0.63	0.36	0.55	
2	Novel CE fabrication	Type 3D-CE P-CE	17.42	0.78	0.68	8.89	162
			14.90	0.78	0.68	7.77	

## Flexible DSSCs. A major innovation in the field

The study of DSSCs has advanced significantly during the past 20 years, with encouraging results. Researchers' interest has recently been drawn to a study of flexible solar cells.^163^ In contrast to stiff glass substrates, DSSC has characteristics such as great flexibility, light weight, and environmental friendliness that make it suitable for these uses.

Employing the doctor blade approach, Sung *et al.*^164^ coated two thin-film samples, namely, a viscous suspension of TiO_2_ and a layer of ZnO on the surface of TiO_2_ powder as a photoelectrode and obtained 1.21%.^163^

The major problem for plastic-type substrates, meanwhile, is temperature, as these materials can resist only 150 °C before melting. The lower temperature during this procedure results in poor nanoparticle connectivity and higher electrode resistance and reduce the performance of plastic-based DSSC. Doctor blades, electrophoretic deposition, hydrothermal, the peel-and-stick method, and pulse laser deposition are under study for obtaining more efficient flexible DSSCs. The most recent technique involves a further step employing titanium(iv) tetraisopropoxide or UV-O_3_ treatment in the manufacture of flexible DSSC.^164^

## DSSC-related issues and their possible solution

Numerous studies have been conducted in DSSC to improve the cell's efficacy and viability. Regardless, the NDSSC's competency is inferior to that of synthetic DSSC for the following reasons. (i) In comparison to silicon crystalline solar cells, DSSCs are inefficient. Using proper components, *e.g.*, dye, substrate, and electrolyte, can likely improve it. Today, the incorporation of graphene into DSSCs increases the PCE of the cells. (ii) When solar radiation is present, dye degradation causes problems with DSSC formation. This hurdle can be overcome by identifying sensitizers with adhesion at a range of temperatures. The synthetic dye, represented by ruthenium structures, is more stable than natural colors because it degrades less in sunlight. The improved proficiency of a cell may be indicated by the presence of dyes that have been mixed. (iii) The use of a liquid electrolyte raises concerns regarding temperature stability. The electrolyte solidifies at low temperatures and expands at high temperatures, which can be devastating to the cell. Thus, solid electrolyte DSSCs are currently being researched. Regardless, the proficiency of the cell is lowered. (iv) In comparison to silicon solar cells, the DSSC exhibits low absorption in the red portion of the sun spectrum, resulting in a restricted current age *via* DSSC. This issue of DSSCs can be resolved through sensitizer enhancements. Sensitized dye has a lower ingestion rate than the characteristic dye. The challenges obstruct large-scale manufacture and widespread adoption of DSSC innovation. Due to the development cycle, DSSCs are still in their infancy and require upgrades to increase their performance and effectiveness. The various findings from this survey article have been summarized below in terms of the improvement obtained in each component of the DSSC.

## Enhancements to the photoanode

While experimenting with various nanostructures for the light electrode, it was determined that the optimum efficiency of 5 to 6.6 was attained by combining graphene layers of numerous diameters. Expanding the width of the graphene sheet varying from 184 nm to 1.2 mm reduces the productivity, demonstrating that a nanostructure with a thinner layer should be preferred. Examining the seed layer of the photoelectrode revealed that fabricating the DSSC with the graphene–titania film resulted in a high degree of proficiency in the region of 3–5% with a fill factor (FF) of about 0.6.

### (A) Developments to sensitizers

Regarding several innovative dyes investigated for the fabrication of the photovoltaic devices, it was determined that a surprisingly high efficacy of 5.87 and 6.90% was achieved using D–A–pi–An indoline dye.

### (B) Electrolyte enhancements

According to the research papers examining the electrolyte improvements for the DSSC, it was discovered that by applying various arrangement convergence of copper iodide particles, the efficiency increased from 4.1–5.1%.

### (C) Developments to the counter anode

The incorporation of platinum in counter cathodes and 3D–CE also shown exceptional efficacy in DSSC. Counter anodes created using a novel nanostructure technique have demonstrated a productivity of up to 7.96%. Alterations in the nanostructure of the counter anode can be used to constrain the efficiency of the cells.

## Future research scope

All the above works have appeared convincing enhancement in the working of the DSSC. DSSCs are turning into the fate of vitality on account of their cost adequacy and expanding transformation effectiveness levels. As of now, the single junction DSSC has achieved transformation efficiency gains of over 13% for the first time. If the current pace of progress continues, productivity levels should rise 15% by the time full commercialization is achieved. Aside from mastering new skills, the most difficult tasks lie in improving the apparatus' strength and lowering the prices of materials and assembly. In addition, improving the cell stability over a long period of time is an important topic that should be addressed in the future. The most common cause of instability in DSSCs is the leakage of liquid electrolyte and the deterioration of the Pt catalyst. A good way to avoid spills is to use a solid-state transport material (HTM) in place of fluid electrolyte.

Furthermore, it is essential to reduce material and assembly costs to improve the performance of the equipment. This component is one of DSSC's most extensive, utilizing toxic crude ingredients and necessitating complex treatment. Natural dyes are generally accessible, simple to plan, financially cost effective, nonharmful, environment friendly, and completely biodegradable. There exists an assortment of natural dye and stabilizers; the productivity can be additionally enhanced by settling on a suitable decision of natural dye and co-sensitized it with a stabilizer. There is still extension for further enhancement in the DSSC regarding every one of its parts. The DSSC can be utilized as an adaptable, ease, and condition cordial solution for energy age from the solar cell. With upgrades, the DSSC can fill in as a commercial product item later. It has a genuinely decent potential to be utilized in building incorporated photovoltaics because of the transparent nature. It is foreseen that DSSC innovation can be utilized to address the three future energy needs, including monetary development, energy security, and condition assurance environment protection. The development of quantitative tools is needed to find suitable semiconductors, dyes, and electrolytes to meet these objectives.

## Conclusion

This evaluation summarizes all recent work performed to improve the DSSC's operation. This review can assist scientists in compiling and integrating several of the cited works to produce a superior professional product. Naturally organic dye sensitizers are inexpensive, easily extracted, abundant, and environmentally beneficial. Ruthenium dyes are right now considered as the best dye for the generation of productive DSSC having proficiency of 10–11%. To get much less expensive dye for DSSC, metal-free organic photosensitizes are strongly wanted. Thus, natural dye separated from various effectively accessible bloom and natural products are reasonable options for conceivable application as sensitizers to inorganic dyes in DSSCs. The results are encouraging and can be used to justify additional investigations into the discovery of new natural sensitizers and the development of solar cell components compatible with such a dye. The sensitizer is the dye in DSSCs and should not deteriorate rapidly, hence extending the cell's life. The cumulative effect of the continual improvements can significantly enhance the DSSC's overall power conversion efficiency. DSSC is currently at its early stage, requiring a significant amount of work to achieve excellent effectiveness. DSSCs have a maximum electrical conversion efficiency of 25%, which is still poor in comparison to traditional silicon-based solar cells. In addition, the DSSC's life is a constraint on its growth; hence, real research would be necessary to improve the DSSC's efficiency and life.

## Conflicts of interest

The authors declare that they have no conflict of interest.

## Supplementary Material
